# Hemorrhagic Fever with Renal Syndrome Patients Exhibit Increased Levels of Lipocalin-2, Endothelin-1 and NT-proBNP

**DOI:** 10.3390/life13112189

**Published:** 2023-11-10

**Authors:** Lidija Cvetko Krajinović, Kristian Bodulić, Renata Laškaj, Branka Žibrat, Petra Svoboda Karić, Ivan-Christian Kurolt, Mihaela Kordun, Antea Topić, Rok Čivljak, Tomislava Skuhala, Alemka Markotić

**Affiliations:** 1 University Hospital for Infectious Diseases “Dr. Fran Mihaljević”, 10 000 Zagreb, Croatia; 2 School of Medicine, University of Zagreb, 10 000 Zagreb, Croatia; 3 School of Dental Medicine, University of Zagreb, 10 000 Zagreb, Croatia; 4 Faculty of Medicine, University of Rijeka, 51 000 Rijeka, Croatia; 5 Faculty of Medicine, Catholic University of Croatia, 10 000 Zagreb, Croatia

**Keywords:** HFRS, *Orthohantavirus*, lipocalin-2, endothelin-1, NT-proBNP

## Abstract

Hemorrhagic fever with renal syndrome (HFRS) is an acute zoonotic disease caused by viruses of the *Orthohantavirus* genus. This syndrome is characterized by renal and cardiopulmonary implications detectable with different biomarkers. Here, we explored the role of serum and urine levels of lipocalin-2, endothelin-1 and N-terminal pro-brain natriuretic peptide (NT-proBNP) in HFRS pathology. A total of twenty-eight patients hospitalized due to a Puumala orthohantavirus infection were included, with serum and urine samples collected on patient admission (acute phase) and discharge (convalescent phase). In comparison to healthy individuals, patients exhibited significantly higher acute-phase serum and urine levels of lipocalin-2, serum levels of endothelin-1 and serum and urine levels of NT-proBNP. Patients in the convalescent phase showed a significant decrease in urine lipocalin-2, serum endothelin-1 and serum and urine NT-proBNP levels. We recorded a strong correlation between serum levels of lipocalin-2 and endothelin-1 and urine levels of lipocalin-2 with several kidney injury markers, such as serum creatinine, urea, urine white blood cell count and proteinuria. We also demonstrated an independent correlation of serum and urine lipocalin-2 levels with acute kidney injury in HFRS. All in all, our results show an involvement of NT-proBNP, lipocalin-2 and endothelin-1 in the renal and cardiac pathology of HFRS.

## 1. Introduction

Hemorrhagic fever with renal syndrome (HFRS) is a zoonotic disease caused by members of the *Orthohantavirus* genus. This genus encompasses enveloped negative-sense single-stranded RNA viruses mainly transmitted by rodents. Cases of transmission to humans occur via infectious aerosols from rodent excreta and have been recorded worldwide, with Puumala orthohantavirus (PUUV) and Dobrava orthohantavirus (DOBV) being the most prominent species in Europe [1,2]. Outbreaks of the PUUV and DOBV viruses are relatively common in Central and Northern Europe, with a few thousand registered cases annually [3]. Consequently, these viruses represent an ongoing public health threat.

The clinical course of HFRS is largely dependent on the viral species causing the disease. For instance, patients infected with PUUV usually present with mild to moderate disease severity with a relatively low death rate (0.1%) [4]. On the other hand, patients infected with DOBV display moderate to severe disease severity and a higher death rate (6–15%) [5]. Patients usually exhibit general symptoms, including fever, headache, myalgia, back and abdominal pain, acute myopia and nausea. However, severe cases can result in indications such as vascular leakage, cardiac manifestations and acute kidney failure. Laboratory findings characteristic of HFRS include thrombocytopenia and leukocytosis, as well as elevated levels of C-reactive protein (CRP), urea and creatinine. Liver transaminases are usually moderately elevated as well [6,7,8,9].

The course of HFRS is mainly characterized by endothelium dysfunction manifesting in increased vascular permeability, which can lead to hypotension, vascular leakage and edema formation [10]. At the same time, immunopathological events accompanying the disease progression include activation of innate and specific immunity mechanisms, e.g., induction of type 1 interferons. These events upregulate the production of various pro-inflammatory cytokines that are associated with the symptoms and possible complications of the disease [11,12,13].

One of the common hallmarks of HFRS is acute kidney injury (AKI). Complications that accompany AKI include proteinuria, hematuria, lowered glomerular filtration rate (GFR) and, in severe cases, kidney failure [6,7,8,9]. Kidney damage is mostly caused by endothelium dysfunction, local infiltration of white blood cells (WBCs) and an upregulated local production of pro-inflammatory cytokines [10]. The events surrounding AKI can usually be detected via various serum and urine biomarkers. For instance, HFRS patients tend to show higher levels of serum creatinine and urea, ionic imbalance and a smaller urine volume [6,7,8,9,14]. Changes in these parameters are typically caused by a decreased GFR and correlate with patients’ length of hospitalization, weight change and disease severity [14].

Some of the biomarkers that may show pertinent potential in assessing the severity of HFRS include serum and urine lipocalin-2, endothelin-1 and N-terminal pro-brain natriuretic peptide (NT-proBNP). Lipocalin-2 is an important component of innate immunity used as a biomarker for kidney injury. This protein is typically expressed in response to AKI by numerous organs and tissues, while its excretion is fully mediated by glomerular filtration. Therefore, the main predictors of lipocalin-2 serum concentration include the rate of lipocalin-2 production, GFR and the rate of lipocalin-2 tubular reabsorption. Moreover, urine levels of lipocalin-2 are mainly determined by its serum levels and, more importantly, by local lipocalin-2 production in the kidney [15,16,17]. Few researchers have addressed the potential of lipocalin-2 as a possible marker for HFRS. The only study conducted on this topic showed a positive correlation between lipocalin-2 urine levels and HFRS patients’ serum creatinine levels, proteinuria, pulmonary complications, weight loss and length of hospitalization [14].

Endothelin-1 is a vasoconstrictive peptide present in pathological states, mainly produced by the endothelium and smooth muscle tissue. Expression of endothelin-1 is typically upregulated in scenarios such as hypoxia and mechanical stress. [18]. This tends to be the case in various pathological processes in the glomeruli and renal tubules. For instance, there has been a plethora of evidence surrounding the correlation between locally produced endothelin-1 and AKI implications, such as proteinuria and renal tubule damage [19,20,21]. Furthermore, endothelin-1 is commonly involved in cardiac pathology, with observed correlations between endothelin-1 and NT-proBNP serum levels, vascular congestion, chronic heart disease and acute heart failure [22]. Notably, research on the potential use of endothelin-1 as a marker for HFRS has been lacking, with only one study showing a potential correlation between endothelin-1 serum levels and kidney vasculature damage [23].

N-terminal pro-brain natriuretic peptide is produced along with brain natriuretic peptide (BNP), which belongs to the family of peptide hormones that regulate blood pressure homeostasis. Unlike BNP, NT-proBNP is not biologically active. The excretion of natriuretic peptides varies between peptide types, where NT-proBNP is mainly excreted through glomerular filtration. Therefore, the levels of serum and urine NT-proBNP tend to be elevated in patients with cardiac and kidney injuries [24,25,26]. Recent research has shown a potential for using serum NT-proBNP as a prognostic factor for HFRS [27]. Furthermore, a significant correlation has been found between serum levels of NT-proBNP and the levels of CRP, serum creatinine, platelet number, hypotension and the length of hospitalization [27]. To our knowledge, urine concentrations of NT-proBNP have not been studied in the context of HFRS severity.

This study examines the involvement of lipocalin-2, endothelin-1 and NT-proBNP in HFRS, as well as their potential use as biomarkers of HFRS severity. The main advantages of these biomarkers come in the light of their relatively high specificity and sensitivity for cardiac and renal injury, but also in their ability to be quickly and easily detected in early disease stages [15,16,17,19,20,21,24,25,26]. These benefits are extremely important for the clinical care of HFRS patients due to a considerable lack of sensitive biomarkers with an ability for early disease severity prognosis.

## 2. Materials and Methods

### 2.1. Test Subjects

Twenty-eight patients with acute PUUV infection were included in this study. All patients were hospitalized at the University Hospital for Infectious Diseases “Dr. Fran Mihaljević” in Zagreb, Croatia, and recruited for the study during the 2012 HFRS outbreak. Puumala virus infection was confirmed via specific RT-PCR and/or serological tests. Acute-phase serum and urine samples were taken routinely on patient admission, while convalescent-phase serum and urine samples were taken on patient discharge. Sera and urine were also collected from fourteen healthy age- and sex-matched individuals and served as a healthy control group. All subjects gave their informed consent for inclusion in the study. This study was conducted in accordance with the Declaration of Helsinki, and the protocol was approved by the Ethics Committee of the University Hospital for Infectious Diseases “Dr. Fran Mihaljević”.

### 2.2. Laboratory Measurements

Lipocalin-2 and endothelin-1 levels were measured using Quantikine ELISA immunoassay (R&D Systems, Minneapolis, MN, USA) according to the manufacturer’s protocol. Serum and urine levels of NT-pro-BNP were measured using an electrochemiluminescence immunoassay (ECLIA) on a Cobas e411 analyzer (Hitachi, Roche Diagnostics, Basel, Switzerland) according to the manufacturer’s protocol. The detectable threshold for NT-proBNP levels was 5 pg/mL.

Complete blood count and hematological and urine parameters were taken routinely. Clinical and laboratory findings were retrospectively collected from patients’ medical records, following standard measures for the protection of subjects’ personal data.

### 2.3. Statistical Analysis

Statistical analysis was performed using R (version 4.2.1., R Core Team, Vienna, Austria) with ggplot2 and corrplot packages [28]. Distribution normality was assessed graphically and with the Shapiro–Wilk test. Numerical variables were reported using medians, interquartile ranges and ranges. Confidence intervals for proportions were calculated using the Clopper–Pearson method. Paired data were compared using the Wilcoxon signed-rank test, while unpaired data were compared using the Mann–Whitney U test. Pairwise correlations between numerical variables were analyzed using the correlation test and Spearman’s rank coefficient. Multivariate linear regression was used to assess the independent correlation of the analyzed biomarkers with maximal serum creatinine during hospitalization. The best model was chosen with the best subset selection method. Predictors and the independent variable were logarithmically scaled. All tests were two-sided with a significance level of 95%. In cases of multiple pairwise testing, *p*-values were adjusted using the Bonferroni method.

## 3. Results

### 3.1. Patients’ Characteristics

A total of twenty-eight HFRS patients were included in this study. The patients’ mean age was 41.15 years (range 25–77 years) and 24 (85.7%) of the patients were male. The patients’ clinical and laboratory parameters are given in Table 1. The most common clinical findings included fever (100.0%), headache (96.4%), proteinuria (89.3%) and myalgia (89.3%). Acute-phase serum and urine were collected upon hospital admission (median day of disease 5, range 3–11), while convalescent-phase serum and urine were collected upon discharge (median day of disease 17.5, range 12–27). None of the patients suffered from renal-associated comorbidities. Seventeen patients (60.7%) exhibited abnormal electrocardiogram (ECG) records during the acute phase of the disease. Sinus bradycardia was the most frequent ECG disorder (N = 5), followed by sinus tachycardia (N = 4), left axis deviation (N = 3), prolonged QT intervals (N = 3) and right bundle branch blocks (N = 2). Every patient included in this study recovered completely.

### 3.2. Lipocalin-2, Endothelin-1 and NT-proBNP Serum and Urine Levels in HFRS

**Figure 1:**
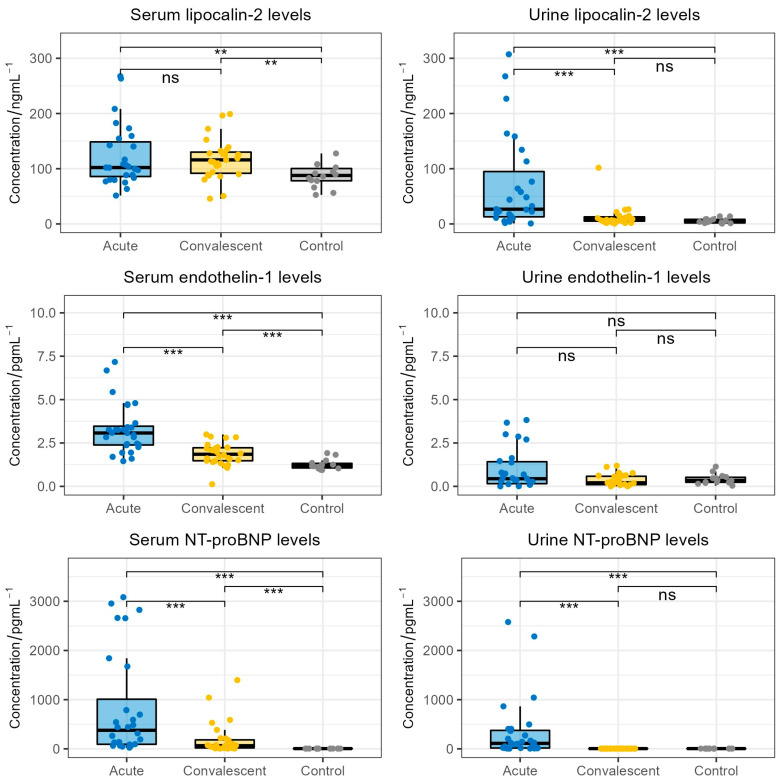
Comparison of lipocalin-2, endothelin-1 and NT-proBNP concentrations in the acute-phase serum and urine (collected on patients’ admission), convalescent-phase serum and urine (collected on patients’ discharge) and serum and urine of the healthy control cohort. The boxes show the median and interquartile range of the distribution, while the whiskers extend to the minimum and maximum non-outlier values of the distribution (ns: non-significant; **: *p* < 0.01; ***: *p* < 0.001; Wilcoxon signed-rank test and Mann–Whitney U test). NT-proBNP = N-terminal pro-brain natriuretic peptide.

The comparison between serum and urine lipocalin-2, endothelin-1 and NT-proBNP levels in HFRS patients and the healthy control cohort is shown in Figure 1. Patients exhibited significantly higher acute-phase (median 102.9 ng/mL) and convalescent-phase (median 116.0 ng/mL) lipocalin-2 serum levels when compared to healthy individuals (median 87.8 ng/mL, *p* = 0.022, *p* = 0.027, respectively). However, we did not observe a significant difference between acute-phase and convalescent-phase serum lipocalin-2 levels (*p* > 0.05). Furthermore, patients in acute HFRS exhibited significantly higher urine lipocalin-2 levels (median 29.5 ng/mL) when compared to patients in the convalescent phase of HFRS (median 8.8 ng/mL, *p* < 0.001) and the healthy control group (median 4.9 ng/mL, *p* < 0.001). Patients in the convalescent phase did not show significant differences in urine lipocalin-2 levels when compared to healthy individuals (*p* > 0.05).

When considering endothelin-1, we observed significantly higher serum endothelin-1 levels in HFRS patients during the acute phase (median 3.1 pg/mL) and the convalescent phase (median 1.9 pg/mL) when compared to healthy individuals (median 1.2 pg/mL, *p* < 0.001, *p* < 0.001, respectively). Moreover, patients in the convalescent phase showed significantly lower serum endothelin-1 levels when compared to patients in the acute phase (*p* < 0.001). On the contrary, we did not observe significant differences in urine endothelin-1 levels between HFRS patients and healthy individuals.

When analyzing NT-proBNP levels, we found significantly higher serum NT-proBNP levels in acute-phase patients (median 377.7 pg/mL) and convalescent-phase patients (median 64.0 pg/mL) when compared to healthy individuals (median 5 pg/mL, *p* < 0.001, *p* < 0.001, respectively). Furthermore, patients in the acute phase exhibited significantly higher serum NT-proBNP levels than patients in the convalescent phase (*p* < 0.001). Patients also exhibited significantly higher acute-phase urine NT-proBNP levels (median 112.0 pg/mL) compared to convalescent-phase urine NT-proBNP levels (median 5 pg/mL, *p* < 0.001) and the urine NT-proBNP levels of healthy individuals (median 5 pg/mL, *p* < 0.001). Notably, the convalescent-phase NT-proBNP levels were extremely similar to the NT-proBNP levels of the healthy control group.

Finally, we directly compared the levels of NT-proBNP, lipocalin-2 and endothelin-1 in the sera and urine of the acute HFRS patients. We found that the serum levels of lipocalin-2, endothelin-1 and NT-proBNP were significantly increased in comparison to the urine levels of the respective biomarker (lipocalin-2 and endothelin-1: *p* < 0.001, NT-proBNP: *p* = 0.007).

### 3.3. Correlation between Serum and Urine Levels of Lipocalin-2, Endothelin-1 and NT-proBNP

**Figure 2:**
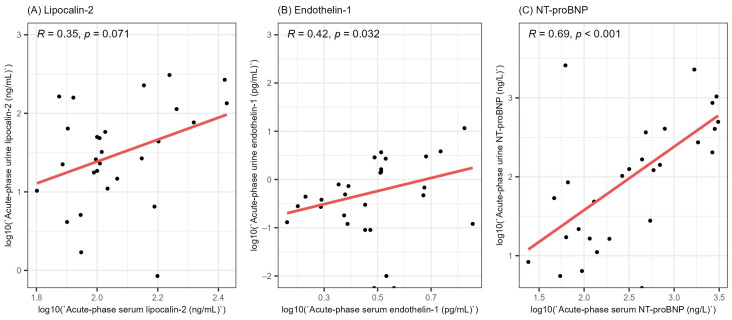
Correlation between the acute-phase serum and urine levels of lipocalin-2 (**A**), endothelin-1 (**B**) and NT-proBNP (**C**). All values were logarithmically transformed before plotting to ensure better visualization. R = Spearman’s correlation coefficient, NT-proBNP = N-terminal pro-brain natriuretic peptide.

In order to gain a better understanding of the involvement of lipocalin-2, endothelin-1 and NT-proBNP in HFRS, we evaluated the correlation between the acute-phase serum and urine levels of the stated biomarkers (Figure 2). We did not find a significant correlation between the acute-phase serum and urine levels of lipocalin-2 (R = 0.35, *p* = 0.071). On the other hand, we found a weak positive correlation between the serum and urine levels of endothelin-1 (R = 0.42, *p* = 0.032) and a strong correlation between the acute-phase serum and urine levels of NT-proBNP (R = 0.69, *p* < 0.001).

### 3.4. Correlation of Lipocalin-2, Endothelin-1 and NT-proBNP Levels with HFRS Severity

**Figure 3:**
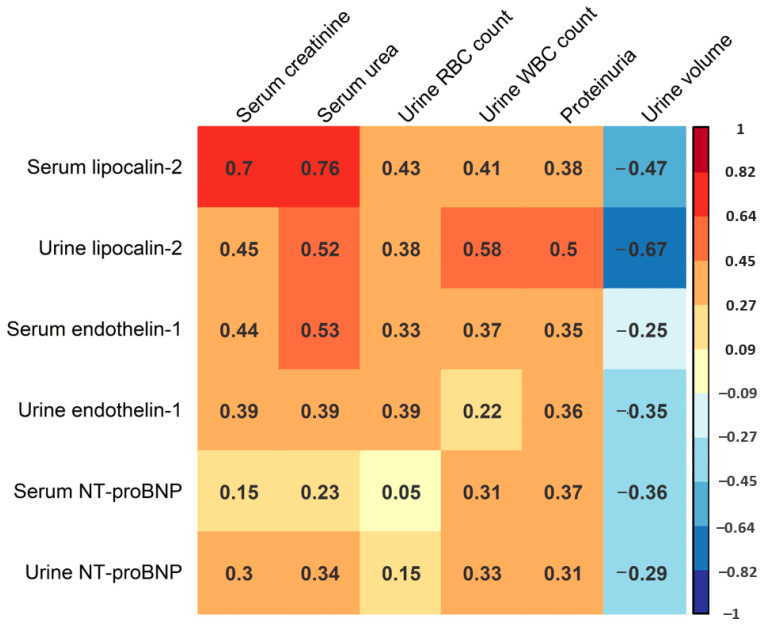
Correlation matrix of acute-phase serum and urine lipocalin-2, endothelin-1 and NT-proBNP levels and the maximal levels of serum creatinine and urea, maximal urine RBC and WBC count, most severe proteinuria and minimal urine volume during hospitalization. Presented values correspond to Spearman’s correlation coefficient. Color intensity is proportional to the Spearman correlation coefficient as shown in the figure legend.

In the following part of the study, we sought to assess the potential correlation of serum and urine levels of lipocalin-2, endothelin-1 and NT-proBNP with AKI severity in HFRS. This analysis mostly included laboratory parameters correlating with AKI severity, including maximal serum creatinine and urea levels during hospitalization, maximal urine WBC and red blood cell (RBC) count during hospitalization, as well as the most severe proteinuria levels and minimal urine volume during hospitalization (Figure 3). We found a strong positive correlation between serum lipocalin-2 levels and maximal serum creatinine (R = 0.70, *p* < 0.001) and urea (R = 0.76, *p* < 0.001), and a moderate positive correlation between serum lipocalin-2 levels and urine RBC counts (R = 0.43, *p* = 0.031). We also found a positive correlation between urine lipocalin-2 levels and urine WBC count (R = 0.58, *p* < 0.001), and a strong negative correlation between urine lipocalin-2 levels and minimal urine volume (R = −0.67, *p* < 0.001). Additionally, we recorded a moderate positive correlation between urine lipocalin-2 levels and serum creatinine (R = 0.45, *p* = 0. 002), urea (R = 0.52, *p* < 0.001) and proteinuria (R = 0.50, *p* = 0.001). Furthermore, we observed a moderate positive correlation between serum endothelin-1 levels and serum creatinine levels (R = 0.44, *p* = 0.003), as well as serum urea levels (R = 0.53, *p* < 0.001). On the contrary, we did not detect a significant correlation between urine endothelin-1 levels and any of the stated AKI biomarkers (*p* > 0.05), or between serum and urine NT-proBNP levels and any of the analyzed AKI biomarkers (*p* > 0.05). When considering ECG records, patients with ECG abnormalities exhibited higher acute-phase serum (medians 440.5 and 139.0 pg/mL) and urine (medians 141.5 and 85.1 pg/mL) levels of NT-proBNP. However, these differences were not statistically significant (*p* = 0.142 and *p* = 0.335, respectively).

We also used multiple linear regression to assess the correlation of acute-phase serum and urine levels of lipocalin-2, endothelin-1 and NT-proBNP with AKI severity in a multivariate environment. Maximal serum creatinine levels during hospitalization were used as the AKI severity measurement. The model that most accurately predicted maximal serum creatinine levels incorporated the levels of acute-phase serum and urine lipocalin-2. This model suggests a significant positive correlation between acute-phase serum lipocalin-2 levels and maximal serum creatinine levels during hospitalization (an average of 2.68% increase in creatinine titer levels per 1% increase in serum lipocalin-2 levels, *p* < 0.001). Importantly, this model also implies an independent positive correlation between acute-phase urine lipocalin-2 levels and maximal serum creatinine levels (an average of 1.11% increase in creatinine titer levels per 1% increase in urine lipocalin-2 levels, *p* = 0.009). The stated combination of acute-phase serum and urine lipocalin-2 levels explained 84.2% of the variance in the maximal serum creatinine levels (adjusted R^2^ = 0.842).

## 4. Discussion

This study aimed to explore the involvement of lipocalin-2, endothelin-1 and NT-proBNP in HFRS pathology. This syndrome is characterized by vascular leakage and immunopathological events, both of which contribute to AKI and cardiopulmonary involvement [10,11,12,13]. Various biomarkers could play an important part in predicting the clinical course of the disease. Numerous studies have shown a high potential of lipocalin-2 [15,16,17], endothelin-1 [19,20,21,22] and NT-proBNP [24,25,26] in predicting the severity of AKI and cardiac complications, which is why these biomarkers represent potential candidates for usage in HFRS patients’ monitoring.

The significant increase of serum lipocalin-2 levels during HFRS represents a novel finding and implies a possible importance of lipocalin-2 in HFRS pathology. Interestingly, patients did not exhibit a significant drop in serum lipocalin-2 levels after hospital discharge, possibly implying the persistence of proinflammatory effects in the convalescent phase of HFRS. We also recorded significantly higher urine lipocalin-2 levels in acute-phase patients when compared to convalescent-phase patients and healthy individuals, which is in concordance with the results of a similar study and points towards immunopathological events in renal tubules during acute HFRS [14]. When considering endothelin-1 levels, we found a significant increase in acute-phase endothelin-1 serum levels when compared to convalescent-phase and healthy individuals’ endothelin-1 levels. This finding also agrees with the results of a previous study demonstrating increased endothelin-1 levels in HFRS and could be a result of both cardiac and renal endothelium dysfunction in HFRS [23]. On the other hand, the studied patients did not exhibit significant differences in urine endothelin-1 levels when compared to healthy individuals, implying a lack of endothelin-1 involvement in AKI in most of the analyzed patients. However, this finding should be further explored on a larger cohort considering that several patients exhibited higher acute-phase urine endothelin-1 levels than healthy individuals. When analyzing NT-proBNP levels, we found considerably higher serum and urine NT-proBNP levels in acute-phase HFRS when compared to convalescent-phase HFRS and the healthy cohort. While significantly higher serum NT-proBNP levels are in accordance with the results of a similar study [27] and imply significant cardiac events during HFRS, significantly higher urine NT-proBNP levels in acute HFRS represent a novel finding. Furthermore, we found that the serum levels of lipocalin-2, endothelin-1 and NT-proBNP were significantly higher than the levels of their urine counterparts, which is in line with the results of studies on several other diseases [29,30,31].

The moderate positive correlation between acute-phase serum and urine NT-proBNP levels could be a direct consequence of urine NT-proBNP concentrations being mainly dependent on NT-proBNP production and excretion rate, which has already been shown in studies of several other diseases [24,25]. Interestingly, serum NT-proBNP levels did not show a significant correlation with most of the AKI markers, including maximum serum creatinine and the urea levels or proteinuria, which is in direct contrast with a previous study on the role of NT-proBNP in HFRS [27]. The main cause of the increased acute-phase serum and urine NT-proBNP levels in HFRS patients may lie in cardiopulmonary complications caused by this syndrome, which is supported by varying ECG abnormalities during the acute phase of the disease. This could consolidate both serum and urine NT-proBNP as potential biomarkers for the cardiac pathology of HFRS.

In contrast to NT-proBNP, lipocalin-2 did not show a strong correlation between its serum and urine concentrations, which could imply local production of lipocalin-2 by renal tissue and invading WBCs. This assumption is further supported by the results of several studies of other diseases in transgenic animal models and humans [15,16,17,19,20,21]. Another piece of evidence that supports the given argument is a relatively strong correlation between acute-phase urine levels of lipocalin-2 and several AKI parameters, such as serum creatinine and urea levels, minimal urine volume and the number of WBCs in the urine. We also found a moderate correlation between urine lipocalin-2 levels and proteinuria, which agrees with the results of a previous study on lipocalin-2 role in HFRS [14].

The moderate positive correlation between acute-phase serum endothelin-1 levels and biomarkers associated with AKI is in line with the results of a previous study on serum endothelin-1 levels in HFRS patients [23]. Furthermore, increased serum endothelin-1 levels could be a result of the endothelium dysfunction and cardiac pathology characteristic of HFRS, which are implied by patients’ ECG abnormalities and increased levels of acute-phase NT-proBNP. On the other hand, urine endothelin-1 levels were not correlated with commonly used AKI markers such as maximum serum creatinine and urea, which goes hand in hand with the result of insignificant differences between endothelin-1 urine levels in HFRS patients and the healthy control cohort. All in all, more research on the role of endothelin-1 in HFRS pathology is needed.

Using multiple linear regression, we demonstrated that the combination of acute-phase serum and urine levels of lipocalin-2 is effective in predicting the maximal levels of serum creatinine in the analyzed patients. Serum creatinine was chosen as it is one of the most widely used estimators of GFR and AKI severity. Moreover, the regression model demonstrated that serum and urine lipocalin-2 levels were more effective in predicting AKI severity than endothelin-1 or NT-proBNP. The independent correlation of both serum and urine lipocalin-2 levels with AKI severity goes hand in hand with the hypothesis of independent local production of lipocalin-2 in the kidney as a result of local inflammatory processes and the renal pathology of HFRS.

We acknowledge several limitations of this study. First, acute-phase and convalescent-phase samples were not taken on the same disease day, which potentially increased the variance in measured biomarkers’ levels. Additionally, the sample size was relatively low, therefore limiting the statistical power of this study. However, the number of patients was sufficient for recording multiple statistically significant results, pointing towards several novel findings.

In conclusion, acute-phase serum and urine lipocalin-2 and NT-proBNP, as well as serum endothelin-1, seem to be effective markers of HFRS severity, possibly incorporating both renal and cardiac involvement in this syndrome. However, more research is needed to quantify the prognostic ability of these parameters in the clinical setting, as well as to elucidate their potential role in the pathology of HFRS.

## Figures and Tables

**Table 1:** Clinical and laboratory parameters of the analyzed patients.

Parameter	Number of Patients (%, 95% CI)		
Fever	28 (100.0, 87.7–100.0)		
Headache	27 (96.4, 81.7–100.0)		
Proteinuria	25 (89.3, 71.8–97.8)		
Myalgia	25 (89.3, 71.8–97.8)		
Nausea	19 (67.9, 47.7–84.1)		
ECG abnormalities	17 (60.7, 40.6–78.5)		
Hepatomegaly	15 (53.6, 33.9–72.5)		
Abdominal pain	11 (39.3, 21.5–59.4)		
Hyperpyrexia	8 (28.6, 13.3–48.7)		
Hypotension	6 (21.4, 8.3–41.0)		
Oliguria	3 (10.7, 2.3–28.2)		
Parameter	Median	IQR	Range
Length of hospitalization (days)	12.5	10–14	8–23
Duration of fever (days)	7	5.75–8	4–13
Day of disease of acute-phase sample collection	5	4–6.25	3–11
Day of disease of convalescent-phase sample collection	17.5	15.75–19.25	12–27
Maximal serum RBC count (×10^12^/L)	5.05	4.6–5.3	3.90–6.13
Minimal platelet count (×10^9^/L)	48.50	35.5–67.25	16.01–166.02
Maximal sedimentation rate (mm/h)	35	27–50.25	10–68
Maximal serum WBC count (×10^9^/L)	11.20	8.85–12.75	6.22–23.37
Maximal CRP (mg/L)	80.5	48.75–122.5	20.0–178.0
Maximal creatinine (µmol/L)	269	146–374.5	82–1238
Maximal urea (mmol/L)	13.6	8.25–18.05	4.6–35.1
Minimal urine volume (mL/day)	1050	600–1400	0–1650
Maximal urine RBC count (RBC/HPF)	10	5–15	1–30
Maximal urine WBC count (WBC/HPF)	5.5	2–10	2–30

RBC = red blood cells, WBC = white blood cells, CRP = C-reactive protein, HPF = high-power field.

## Data Availability

The data presented in this study are available on the request from the corresponding author.
